# Analysis of risk factors of postoperative complication for non-small cell lung cancer

**DOI:** 10.1186/s12890-024-03054-1

**Published:** 2024-07-10

**Authors:** Nozomu Motono, Takaki Mizoguchi, Masahito Ishikawa, Shun Iwai, Yoshihito Iijima, Hidetaka Uramoto

**Affiliations:** Department of Thoracic Surgery, Kanazawa Medical University, 1- 1 Daigaku, Uchinada, Ishikawa 920-0293 Japan

**Keywords:** Postoperative complication, Non-small cell lung cancer, Body mass index, Chronic obstructive pulmonary disease, Operation time, Prognostic nutrition index

## Abstract

**Background:**

The relationship between risk factors of common postoperative complications after pulmonary resection, such as air leakage, atelectasis, and arrhythmia, and patient characteristics, including nutritional status or perioperative factors, has not been sufficiently elucidated.

**Methods:**

One thousand one hundred thirty-nine non-small cell lung cancer patients who underwent pulmonary resection were retrospectively analyzed for risk factors of common postoperative complications.

**Results:**

In a multivariate analysis, male sex (*P* = 0.01), age ≥ 65 years (*P* < 0.01), coexistence of chronic obstructive pulmonary disease (COPD) (*P* < 0.01), upper lobe (*P* < 0.01), surgery time ≥ 155 min (*P* < 0.01), and presence of lymphatic invasion (*P* = 0.01) were significant factors for postoperative complication. Male sex (*P* < 0.01), age ≥ 65 years (*P* = 0.02), body mass index (BMI) < 21.68 (*P* < 0.01), coexistence of COPD (*P* = 0.02), and surgery time ≥ 155 min (*P* = 0.01) were significant factors for severe postoperative complication. Male sex (*P* = 0.01), BMI < 21.68 (*P* < 0.01), thoracoscopic surgery (*P* < 0.01), and surgery time ≥ 155 min (*P* < 0.01) were significant risk factors for postoperative air leakage. Coexistence of COPD (*P* = 0.01) and coexistence of asthma (*P* < 0.01) were significant risk factors for postoperative atelectasis. Prognostic nutrition index (PNI) < 45.52 (*P* < 0.01), lobectomy or extended resection more than lobectomy (*P* = 0.01), and surgery time ≥ 155 min (*P* < 0.01) were significant risk factors for postoperative arrhythmia.

**Conclusion:**

Low BMI, thoracoscopic surgery, and longer surgery time were significant risk factors for postoperative air leakage. Coexistence of COPD and coexistence of asthma were significant risk factors for postoperative atelectasis. PNI, surgery time, and surgical procedure were revealed as risk factors of postoperative arrhythmia. Patients with these factors should be monitored for postoperative complication.

**Trial registration:**

The Institutional Review Board of Kanazawa Medical University approved the protocol of this retrospective study (approval number: I392), and written informed consent was obtained from all patients.

## Background

The incidence of postoperative complications associated with pulmonary resection for non-small cell lung cancer (NSCLC) was reported to be 9–37% [1–3]. Furthermore, the incidence of postoperative complications associated with lobectomy was 10–50% and was higher in older patients [4]. Several postoperative complications might occur after pulmonary resection, and air leakage, pneumonia, atelectasis, and arrhythmia are considered common complications. In these reports, the incidence of postoperative pulmonary complications after pulmonary resection was 6–30%. Age, smoking history, and chronic obstructive pulmonary disease (COPD) are considered significant risk factors for postoperative pulmonary complications [5–9].

In our previous study, we reported that the coexistence of asthma, neutrophil-to-lymphocyte ratio (NLR), and pulmonary lobe were significant risk factors for postoperative complications [10]. Recently, low body mass index (BMI) and nutritional status, such as prognostic nutrition index (PNI), have been demonstrated as risk factors of postoperative pulmonary complications [11–13]. Furthermore, Charlson comorbidity index (CCI), surgical procedure, and surgery time were showed as risk factors of postoperative complications [14]. However, the relationship between risk factors of common postoperative complications, such as air leakage, atelectasis, and arrhythmia, after pulmonary resection and patient characteristics, including nutritional status, or perioperative factors have not been adequately elucidated.

In the present study, we retrospectively analyzed the risk factors of postoperative complications in NSCLC patients who underwent pulmonary resection, including risk factors of common postoperative complications.

## Methods

### Patients

One thousand one hundred thirty-nine NSCLC patients who underwent pulmonary resection at Kanazawa Medical University between January 2002 and December 2021 were enrolled in this retrospective study.

Data collected were sex, age, smoking history, BMI, comorbidity, CCI, carcinoembryonic antigen (CEA) levels, PNI, NLR, cancer inflammation prognostic index (CIPI), and pulmonary lobe involvement in lung cancer. Smoking history was assessed using the Brinkman Index, which is calculated by multiplying the number of cigarettes smoked per day by the number of years the subject has been smoking [15]. CCI was calculated by score according to the comorbidity grade [16]. Furthermore, comorbidity was divided into six categories: atrial fibrillation, pectoris angina, interstitial lung disease (ILD), COPD, asthma, and diabetes mellitus. PNI was calculated by combining serum albumin levels with the total peripheral lymphocyte count in peripheral blood [17]. NLR was defined as the ratio of neutrophil-to-lymphocyte count, and CIPI was calculated as CEA (ng/ml) × NLR [18, 19].

### Surgical factors

Data collected were surgical approach, surgery time, and surgical procedure. The surgical approach was divided into three categories: robotic-assisted thoracic surgery (RATS), video-assisted thoracic surgery (VATS), and open thoracotomy. We decided to undertake RATS and VATS as the thoracoscopic surgery. The surgical procedure was divided into seven categories: wedge resection, segmentectomy, lobectomy, sleeve lobectomy, lobectomy combined with segmentectomy, lobectomy combined with chest wall resection, bilobectomy, and pneumonectomy.

### Pathological factors

Data on lymphatic invasion, vascular invasion, differentiation, histological type, and pathological stage were collected.

### Postoperative complications

Postoperative complications were categorized into five grades according to the Clavien–Dindo classification system: grade I, II, IIIa, IIIb, IVa, IVb, and V. The suffix “d” (for “disability”) was used to denote any postoperative impairment [20]. Severe complications were defined as grade IIIa or more. Furthermore, we also divided postoperative complications into nine categories for the multivariate analysis of risk factors: air leakage, arrhythmia, atelectasis, pneumonia, chylothorax, home oxygen therapy, cerebral infarction, empyema or pleuritis, and surgical site infection.

### Statistical analyses

Qualitative variables are expressed as absolute numbers and percentages, and quantitative data are expressed as medians and ranges. The cutoff values for factors associated with postoperative complications were calculated using receiver operating characteristic (ROC) curve analysis, and risk analyses were performed using these cutoff values. The univariate and multivariate analysis of the risk factors for the postoperative complications, including severe postoperative complication and each postoperative complications, were performed by logistic regression analysis Multivariate analysis was performed for the factors that showed significant differences in univariate analysis. All statistical analyses were two-sided and statistical significance was set at *P* < 0.05. Statistical analyses were performed using JMP software v14 (SAS Institute Inc., Cary, NC, USA).

## Results

### Patients’ characteristics

The clinicopathological characteristics of the 1139 patients are shown in Table 1. There were 701 men, and the median age was 70.7 years. Median Brinkman Index was 600 and median BMI was 22.71. In total, 682 patients had comorbidities, including 28 patients with atrial fibrillation, 91 with angina pectoris, 41 with ILD, 399 with COPD, 38 with asthma, and 169 with diabetes mellitus. Median CEA concentration was 3.4 ng/ml, median PNI was 49.8, NLR was 2.22, and median CIPI was 7.92. The pulmonary lobes resected for NSCLC included the right upper lobe in 344 patients, right middle lobe in 69, right lower lobe in 271, left upper lobe in 267, and left lower lobe in 188.

**Table 1:** Patient characteristics

Variables	Values
Gender (male / female)	701 / 438
Age, median (range) (y)	70.7 (22–92)
Brinkman index, median (range)	600 (0–3600)
Body mass index, median (range)	22.71 (13.54–41.06)
Comorbidity, present (%)	682 (60%)
Charlson comorbidity index (0 / 1 / 2 / 3 / 4 / 5 / 6 / 7 / 8)	604 / 277 / 192 / 46 / 14 / 3 / 2 / 1
Atrial fibrillation, present (%)	28 (2.5%)
Angina pectoris, present (%)	91 (8.0%)
Interstitial lung disease, present (%)	41 (3.6%)
Chronic obstructive pulmonary disease, present (%)	399 (35.0%)
Asthma, present (%)	38 (3.3%)
Diabetes mellitus, present (%)	169 (14.8%)
CEA, median (range) (ng/ml)	3.4 (0.5–306.0)
PNI, median (range)	49.8 (26.95–67.59)
NLR, median (range)	2.22 (0.48–24.51)
CIPI, median (range)	7.92 (0.72–1465.74)
Lobe of tumor (RU / RM / RL / LU / LL)	344 / 69 / 271 / 267 / 188
Operative approach (RATS/ VATS/Open)	62 / 398 / 679
Operative procedure (Wed/Seg/Lob/Sleeve Lob/Lob + Seg/Lob + CWR/Bilob/Pneumo)	190 / 115 / 756 / 8 / 8 / 13 / 20 / 29
Operation time, median (range) (min)	169 (26–1149)
Histological type (Ad / Sq /LCNEC / AdSq / Pleo / Carci / Large)	839 / 224 / 30 / 17 / 16 / 7 / 6
Lymphatic invasion, present (%)	391 (34.3%)
Vascular invasion, present (%)	491 (43.1%)
Differentiation (G1 / 2 / 3 / 4)	366 / 570 / 168 / 35
Pathological stage (0 / IA / IB / IIA / IIB / IIIA/ IIIB / IV/ yIA / yIIA)	64 / 605 / 202 / 46 / 112 / 94 / 4 / 3 / 8 / 1
Postoperative complications, present (%)	327 (28.7%)
Clavien-Dindo grade (0 / I / II / IIIa / IIIb / IV / V)	812 / 2 / 129 / 183 / 12 / 0 / 1
Air leakage, present (%)	141 (12.3%)
Arrhythmia, present (%)	64 (5.6%)
Pneumonia, present (%)	36 (3.1%)
Atelectasis, present (%)	32 (2.8%)
Chylothorax, present (%)	9 (0.8%)
Home oxygen therapy, present (%)	7 (0.6%)
Cerebral infarction, present (%)	6 (0.5%)
Empyema or pleuritis, present (%)	6 (0.5%)
Surgical site infection, present (%)	6 (0.5%)
Broncho-pleural fistula, present (%)	3 (0.3%)

CEA; carcinoembryonic antigen, PNI; prognostic nutrition index, NLR; neutrophil-to-lymphocyte ratio, CIPI; cancer-inflammation prognostic index, RU; right upper, RM; right middle, RL; right lower, LU; left upper, LL; left lower, RATS; robotic assisted thoracic surgery, VATS; video assisted thoracic surgery, Open; open thoracotomy, Wed; wedge resection, Seg; segmentectomy, Lob; lobectomy, CWR; chest wall resection, Bilob; bi-lobectomy, Pneumo; pneumonectomy, Ad; adenocarcinoma, Sq; squamous cell carcinoma, LCNEC; large cell neuroendocrine carcinoma, AdSq; adenosquamous cell carcinoma, Pleo; pleomorphic carcinoma, Carci; carcinoid, Large; large cell carcinoma, G; grade of differentiation, y; yield to treatment

### Surgical factors

RATS was performed in 62 patients, VATS in 398, and open thoracotomy in 679. With regards to the surgical procedures, wedge resection was performed in 190 patients, segmentectomy in 115, lobectomy in 756, sleeve lobectomy in 8, lobectomy combined with segmentectomy in 8, lobectomy with chest wall resection in 13, bilobectomy in 20, and pneumonectomy in 29. The median surgery time was 169 min.

### Pathological factors

The histological types were as follows: adenocarcinoma in 839 patients, squamous cell carcinoma in 224, large cell neuroendocrine carcinoma in 30, adenosquamous cell carcinoma in 17, pleomorphic carcinoma in 16, carcinoid in 7, and large-cell carcinoma in 6. Lymphatic invasion was present in 391 patients and vascular invasion in 491. Differentiation was categorized as G1 in 36 patients, G2 in 570, G3 in 168, and G4 in 35. The pathological stages were: stage 0 in 64 patients, IA in 605, IB in 202, IIA in 46, IIB in 112, IIIA in 94, IIIB in 4, IV in 8, yield to treatment (y) IA in 8, and yIIA in 1.

### Postoperative complications

Postoperative complications were observed in 327 patients (28.7%). Clavien–Dindo grade I complications were noted in 2 patients, grade II in 129, grade IIIa in 183, grade IIIb in 12, and grade V in 1. Major postoperative complications included air leakage in 141 patients, arrhythmia in 64, pneumonia in 36, and atelectasis in 32. Minor postoperative complications were chylothorax in nine patients, home oxygen therapy in seven, cerebral infarction in six, empyema or pleuritis in six, surgical site infection in six, and broncho-pleural fistula in three.

### Cutoff values calculated from ROC curves

The cutoff values of factors associated with postoperative complications were calculated by ROC curve analysis. The following cutoff values were determined: age, 65 years; BMI, 21.68; PNI, 45.52; CIPI, 7.25; CIPI, 14.59; and surgery time, 155 min.

### Univariate and multivariate analyses

The relationship between patient characteristics and postoperative complications was analyzed (Table 2). The following were significant factors for postoperative complications in the univariate analysis: male sex (*P* < 0.01), age ≥ 65 years (*P* < 0.01), Brinkman index ≥ 600 (*P* < 0.01), coexistence of COPD (*P* < 0.01), PNI < 45.52 (*P* < 0.01), CIPI > 7.25 (*P* = 0.01), upper lobe (*P* < 0.01), lobectomy or extended resection more than lobectomy (*P* < 0.01), surgery time ≥ 155 min (*P* < 0.01), non-adenocarcinoma (*P* < 0.01), presence of lymphatic invasion (*P* < 0.01), presence of vascular invasion (*P* = 0.02), and presence of grade 3/4 differentiation (*P* < 0.01). Male sex (*P* = 0.01), age ≥ 65 years (*P* < 0.01), coexistence of COPD (*P* < 0.01), upper lobe (*P* < 0.01), surgery time ≥ 155 min (*P* < 0.01), and presence of lymphatic invasion (*P* = 0.01) were significant factors for postoperative complication in the multivariate analysis. The relationship between patient characteristics and severe postoperative complication was analyzed (Table 3). The following were significant factors for severe postoperative complication in the univariate analysis: male sex (*P* < 0.01), age ≥ 65 years (*P* = 0.04), Brinkman index ≥ 600 (*P* < 0.01), BMI < 21.68 (*P* < 0.01), coexistence of COPD (*P* < 0.01), CIPI > 7.25 (*P* < 0.01), lobectomy or extended resection more than lobectomy (*P* = 0.01), surgery time ≥ 155 min (*P* < 0.01), non-adenocarcinoma (*P* = 0.02), presence of lymphatic invasion (*P* = 0.01), and presence of grade 3/4 differentiation (*P* = 0.01). Male sex (*P* < 0.01), age ≥ 65 years (*P* = 0.02), BMI < 21.68 (*P* < 0.01), coexistence of COPD (*P* = 0.02), and surgery time ≥ 155 min (*P* = 0.01) were significant factors for severe postoperative complication in the multivariate analysis.

**Table 2:** Univariate analysis and multivariate analysis of risk factor for postoperative complication

Univariate analysis					Multivariate analysis		
Variables		Odds ratio	95% CI	*P* value	Odds ratio	95% CI	*P* value
Gender	male	2.23	1.682–2.970	< 0.01	1.58	1.083–2.312	0.01
Age	≥ 65	1.59	1.175–2.176	< 0.01	1.59	1.145–2.231	< 0.01
Smoking status	BI ≥ 600	1.94	1.496–2.530	< 0.01	0.99	0.681–1.442	0.96
BMI	< 21.68	1.26	0.974–1.654	0.07			
Charlson comorbidity index	≥ 3	0.65	0.357–1.196	0.16			
Atrial fibrillation	present	0.67	0.269–1.670	0.39			
Angina pectoris	present	1.31	0.833–2.066	0.24			
Interstitial lung disease	present	0.79	0.385–1.640	0.53			
COPD	present	2.02	1.554–2.633	< 0.01	1.57	1.177–2.106	< 0.01
Asthma	present	1.84	0.957–3.564	0.06			
Diabetes mellitus	present	0.949	0.659–1.366	0.77			
PNI	< 45.52	1.52	1.123–2.072	< 0.01	1.27	0.922–1.773	0.14
CIPI	> 7.25	1.40	1.084–1.826	0.01	1.11	0.844–1.485	0.43
Location of lesion	Upper lobe	1.50	1.162–1.960	< 0.01	1.49	1.134–1.970	< 0.01
Operative approach	Thoracoscopic surgery	1.01	0.782–1.320	0.90			
Operative procedure	Lobe or extend	1.77	1.300-2.433	< 0.01	1.34	0.931–1.951	0.11
Operation time	≥ 155 min	2.04	1.558–2.688	< 0.01	1.76	1.281–2.423	< 0.01
Histological type	Non-adenocarcinoma	1.76	1.333–2.336	< 0.01	1.23	0.886–1.707	0.21
Lymphatic invasion	present	1.61	1.237–2.102	< 0.01	1.51	1.077–2.130	0.01
Vascular invasion	present	1.34	1.038–1.740	0.02	0.81	0.580–1.152	0.25
Differentiation	G3-4	1.76	1.286–2.427	< 0.01	1.35	0.942–1.942	0.10
Pathological stage	≥ II	1.15	0.855–1.553	0.34			

CI; confidence interval, BI; Brinkman index, BMI; body mass index, COPD; chronic obstructive pulmonary disease, PNI; prognostic nutrition index, CIPI; cancer-inflammation prognostic index, Lobe or extend; lobectomy or extend resection more than lobectomy, G; grade of differentiation

**Table 3:** Univariate analysis and multivariate analysis of risk factor for postoperative severe complication

Univariate analysis					Multivariate analysis		
Variables		Odds ratio	95% CI	*P* value	Odds ratio	95% CI	*P* value
Gender	male	2.24	1.578–3.194	< 0.01	2.03	1.286–3.233	< 0.01
Age	≥ 65	1.46	1.007–2.122	0.04	1.55	1.048–2.321	0.02
Smoking status	BI ≥ 600	1.74	1.273–2.391	< 0.01	0.87	0.561–1.352	0.53
BMI	< 21.68	1.91	1.402–2.612	< 0.01	2.13	1.541–2.952	< 0.01
Charlson comorbidity index	≥ 3	0.74	0.363–1.537	0.42			
Atrial fibrillation	present	0.79	0.273–2.325	0.67			
Angina pectoris	present	1.11	0.642–1.935	0.69			
Interstitial lung disease	present	0.51	0.179–1.448	0.20			
COPD	present	1.78	1.307–2.440	< 0.01	1.47	1.051–2.079	0.02
Asthma	present	2.01	0.983–4.138	0.05			
Diabetes mellitus	present	1.09	0.717–1.674	0.67			
PNI	< 45.52	1.30	0.902–1.874	0.15			
CIPI	> 7.25	1.52	1.111–2.092	< 0.01	1.19	0.853–1.675	0.29
Location of lesion	Upper lobe	1.24	0.913–1.703	0.16			
Operative approach	Thoracoscopic surgery	1.07	0.786–1.469	0.64			
Operative procedure	Lobe or extend	1.58	1.083–2.308	0.01	1.35	0.871–2.115	0.17
Operation time	≥ 155 min	1.80	1.301–2.511	< 0.01	1.58	1.078–2.316	0.01
Histological type	Non-adenocarcinoma	1.45	1.041–2.026	0.02	0.99	0.680–1.457	0.98
Lymphatic invasion	present	1.47	1.077–2.021	0.01	1.23	0.871–1.739	0.23
Vascular invasion	present	1.23	0.908–1.683	0.17			
Differentiation	G3-4	1.59	1.099–2.304	0.01	1.25	0.832–1.880	0.28
Pathological stage	≥ II	0.96	0.667–1.386	0.83			

CI; confidence interval, BI; Brinkman index, BMI; body mass index, COPD; chronic obstructive pulmonary disease, PNI; prognostic nutrition index, CIPI; cancer-inflammation prognostic index, Lobe or extend; lobectomy or extend resection more than lobectomy, G; grade of differentiation

### Risk factors of each postoperative complication

The significant risk factors of each postoperative complication by multivariate analysis are shown in Table 4. Male sex (*P* < 0.01), age ≥ 65 years (*P* = 0.03), Brinkman index ≥ 600 (*P* = 0.02), BMI < 21.68 (*P* < 0.01), coexistence of COPD (*P* < 0.01), CIPI > 7.25 (*P* = 0.01), thoracoscopic surgery (*P* = 0.01), and surgery time ≥ 155 min (*P* = 0.04) were significant risk factors for postoperative air leakage in the univariate analysis (data not shown). However, male sex (*P* = 0.01), BMI < 21.68 (*P* < 0.01), thoracoscopic surgery (*P* < 0.01), and surgery time ≥ 155 min (*P* < 0.01) were significant risk factors for postoperative air leakage in the multivariate analysis.

**Table 4:** Multivariate analysis of risk factors for each postoperative complication

Postoperative complication	Risk factors		Odds ratio	95%CI	*P* value
Air leakage (*n* = 141)	Gender	Male	1.93	1.156–3.242	0.01
	Age	≥ 65	1.56	0.988–2.437	0.05
	Smoking status	BI ≥ 600	0.82	0.514–1.327	0.43
	BMI	< 21.68	2.31	1.605–3.347	< 0.01
	COPD	Present	1.45	0.988–2.141	0.05
	CIPI	> 7.25	1.29	0.880–1.900	0.18
	Operative approach	Thoracoscopic surgery	1.80	1.236–2.621	< 0.01
	Operation time	≥ 155 min	1.72	1.162–2.563	< 0.01
Arrhythmia (*n* = 64)	PNI	< 45.52	2.17	1.252–3.761	< 0.01
	Operative approach	Thoracoscopic surgery	0.77	0.422–1.415	0.40
	Operative procedure	Lobe or extend	5.99	1.381–26.021	0.01
	Operation time	≥ 155 min	3.10	1.413–6.815	< 0.01
	Histological type	Non-adenocarcinoma	1.55	0.900-2.698	0.11
	Lymphatic invasion	Present	1.08	0.580–2.026	0.79
	Vascular invasion	Present	1.31	0.693-2.500	0.40
	Differentiation	G3-4	1.35	0.719–2.541	0.34
	Pathological stage	≥ II	0.88	0.495–1.579	0.67
Atelectasis (*n* = 32)	COPD	Present	2.46	1.189–5.093	0.01
	Asthma	Present	5.15	1.822–14.564	< 0.01
	Location of lesion	Upper lobe	2.10	0.959–4.638	0.06
Pneumonia (*n* = 36)	Gender	Male	1.23	0.398–3.832	0.71
	Smoking status	BI ≥ 600	2.88	0.960–8.856	0.05
	COPD	Present	1.85	0.918–3.757	0.08
	Histological type	Non-adenocarcinoma	1.16	0.566–2.402	0.67
Chylothorax (*n* = 9)	Not significant				
Home oxygen therapy (*n* = 7)	Not significant				
Cerebral infarction (*n* = 6)	Not significant				
Empyema or pleuritis (*n* = 6)	Not significant				
Surgical site infection (*n* = 6)	Not significant				

CI; confidence interval, BI; Brinkman index, BMI; body mass index, COPD; chronic obstructive pulmonary disease, CIPI; cancer-inflammation prognostic index, PNI; prognostic nutrition index, Lobe or extend; lobectomy or extend resection more than lobectomy, G; grade of differentiation

PNI < 45.52 (*P* < 0.01), thoracoscopic surgery (*P* = 0.01), lobectomy or extended resection more than lobectomy (*P* < 0.01), surgery time ≥ 155 min (*P* < 0.01), non-adenocarcinoma (*P* < 0.01), presence of lymphatic invasion (*P* = 0.03), presence of vascular invasion (*P* = 0.01), presence of grade 3/4 differentiation (*P* = 0.02), and pathological stage ≥ II (*P* = 0.03) were significant factors for postoperative arrhythmia in the univariate analysis (data not shown). In the multivariate analysis, PNI < 45.52 (*P* < 0.01), lobectomy or extended resection more than lobectomy (*P* = 0.01), and surgery time ≥ 155 min (*P* < 0.01) were significant risk factors for postoperative arrhythmia.

Coexistence of COPD (*P* < 0.01), coexistence of asthma (*P* < 0.01), and upper lobe (*P* = 0.04) were significant factors for postoperative atelectasis in the univariate analysis (data not shown). However, coexistence of COPD (*P* = 0.01) and coexistence of asthma (*P* < 0.01) were the only significant risk factors for postoperative atelectasis in the multivariate analysis.

While male sex (*P* < 0.01), Brinkman index ≥ 600 (*P* < 0.01), coexistence of COPD (*P* < 0.01), and non-adenocarcinoma (*P* = 0.03) were significant factors for postoperative pneumonia in the univariate analysis (data not shown), there were no significant risk factors for postoperative pneumonia in the multivariate analysis.

Although we analyzed the risk factors of chylothorax, home oxygen therapy, cerebral infarction, empyema or pleuritis and surgical site infection, significant risk factors could not be identified.

## Discussion

We analyzed risk factors of postoperative complication for NSCLC patients who underwent pulmonary resection, including risk factors of common postoperative complications. Male sex, high age, coexistence of COPD, upper lobe, longer surgery time, and presence of lymphatic invasion were significant factors for postoperative complication. Furthermore, male sex, high age, low BMI, coexistence of COPD, and longer surgery time were significant factors for severe postoperative complication. Age, comorbidity, smoking history, surgical approach, and type of surgical procedure have previously been showed as risk factors for postoperative complication in NSCLC patients who have undergone pulmonary resection [4, 6, 7, 14, 21, 22]. In addition, upper lobe location and surgery time were revealed as risk factors for postoperative complication in this study. The pulmonary lobe of the tumor, including the right upper lobe, right lower lobe, and left upper lobe, was a significant risk factor for postoperative complication in our previous study [10]. Furthermore, longer surgery time has been reported as a risk factor of postoperative complication for lung cancer patients who underwent pulmonary lobectomy [23]. Male sex, high age, coexistence of COPD, and longer surgery time were revealed as risk factors of both postoperative complication and severe postoperative complication in this study; hence, patients with these factors should be especially monitored for postoperative complication, and the effort to shorten the surgery time should be important.

High age and low BMI have been demonstrated as risk factors of postoperative pulmonary complication [12, 13]. Male sex, low BMI, thoracoscopic surgery, and longer surgery time were significant risk factors for postoperative air leakage, and coexistence of COPD and coexistence of asthma were significant risk factors for postoperative atelectasis, while there was no significant risk factor of postoperative pneumonia identified in this study. In a previous study, male sex and thoracoscopic surgery were revealed as risk factors of postoperative air leakage, and the presence of asthma was showed as a risk factor of postoperative atelectasis or pneumonia [24]. Although risk factors of postoperative pneumonia were not revealed in current study, smoking status tended to be a risk factor of postoperative pneumonia and then the careful follow-up should be necessary.

Although surgical procedure has previously been reported as a risk factor of postoperative arrhythmia [24], PNI and surgery time, in addition to surgical procedure, were revealed as risk factors of postoperative arrhythmia in this study. Nutritional status has not been reported as a risk factor of postoperative arrhythmia for NSCLC patients who have undergone pulmonary resection, and further studies are needed. Longer surgery time might induce autonomic denervation and stress-mediated neurohumoral mechanisms resulting postoperative arrythmia. Surgical procedure, such as lobectomy or extended resection more than lobectomy, might induce a large load to the right heart system and cause arrhythmia by reducing pulmonary vasculature.

The utility of sublobar resection for early-stage NSCLC was recently revealed [25–27]. Although sublobar resection is associated with less postoperative complications than lobectomy in these reports, there were no significant differences in risk of postoperative complications among surgical procedures in this study. Postoperative complications by surgical procedures might be significant differences in patient populations such as the elderly or with emphysematous lungs, and the future studies should be needed.

Although chylothorax and cerebral infarction are crucial postoperative complications for NSCLC patients who have undergone pulmonary resection, it might be difficult to statistically determine the risk factors of these postoperative complications because of the small numbers of patients with such complications, as in this study.

This study had several limitations. First, it was a retrospective study, and it potentially involved unobserved cofounding and selection biases. Second, the study was performed at a single institution, and the study population was small. The small representation of atrial fibrillation or asthma or interstitial lung disease in this study might be limited the effect to risk on postoperative complications including postoperative severe complications and each postoperative complication. Third, the possibility cannot be ruled out that there were cases that were not included in this study due to insufficient data, resulting in selection bias.

## Conclusions

We analyzed risk factors of postoperative complication for NSCLC patients who underwent pulmonary resection, including risk factors of common postoperative complications. Male sex, high age, coexistence of COPD, and longer surgery time were revealed as risk factors of both postoperative complication and severe postoperative complication in this study. For each postoperative complication, male sex, low BMI, thoracoscopic surgery, and longer surgery time were significant risk factors for postoperative air leakage, while coexistence of COPD and coexistence of asthma were significant risk factors for postoperative atelectasis, and PNI, surgery time, and surgical procedure were revealed as risk factors of postoperative arrhythmia. There was no significant risk factor of postoperative pneumonia. Patients with these factors should be carefully monitored for each postoperative complication.

## Data Availability

The datasets generated and/or analyzed during the current study are not publicly available due to [our institutional restrictions e.g., them containing information that could compromise research participant privacy/consent], but are available from the corresponding author on reasonable request.

## Abbreviations

NSCLC: Non-small cell lung cancer
COPD: Chronic obstructive pulmonary disease
NLR: Neutrophil-to-lymphocyte ratio
BMI: Body mass index
PNI: Prognostic nutrition index
CCI: Charlson comorbidity index
CEA: Carcinoembryonic antigen
CIPI: Cancer inflammation prognostic index
ILD: Interstitial lung disease
RATS: Robotic-assisted thoracic surgery
VATS: Video-assisted thoracic surgery
ROC: Receiver operating characteristic
